# Ambient awareness: From random noise to digital closeness in online social networks

**DOI:** 10.1016/j.chb.2016.02.037

**Published:** 2016-07

**Authors:** Ana Levordashka, Sonja Utz

**Affiliations:** a Leibniz-Institut für Wissensmedien, Tübingen, Germany; b University of Tübingen, Tübingen, Germany

**Keywords:** Ambient awareness, Social presence, Impression formation, Social media, Social capital, Twitter

## Abstract

Ambient awareness refers to the awareness social media users develop of their online network in result of being constantly exposed to social information, such as microblogging updates. Although each individual bit of information can seem like random noise, their incessant reception can amass to a coherent representation of social others. Despite its growing popularity and important implications for social media research, ambient awareness on public social media has not been studied empirically. We provide evidence for the occurrence of ambient awareness and examine key questions related to its content and functions. A diverse sample of participants reported experiencing awareness, both as a general feeling towards their network as a whole, and as knowledge of individual members of the network, whom they had not met in real life. Our results indicate that ambient awareness can develop peripherally, from fragmented information and in the relative absence of extensive one-to-one communication. We report the effects of demographics, media use, and network variables and discuss the implications of ambient awareness for relational and informational processes online.

## Highlights

• Ambient awareness: Browsing social media enhances familiarity with online networks.
• In 2 surveys, we provide evidence for ambient awareness among Twitter users.
• Frequent users experienced higher ambient awareness.
• Ambient awareness was reflected in knowledge of various domains, such as expertise.
• Ambient awareness was associated with perceptions of approachability.

## 1 Introduction

“What is happening right now?” is a question social media and networking sites constantly ask their users. The typically brief answers are then broadcasted to large audiences, often a person's entire network on the given site. At the same time, people receive and skim through updates from friends, relatives, acquaintances, or even strangers. This type of communication is perhaps best characterized by the incessant flow of brief and mundane bits of information. Closely linked to the ubiquity of social-networking sites and mobile devices that allow people to be permanently online and connected, such incessant mediated communication is unprecedented and scholars are yet to understand its interpersonal effects (Vorderer & Kohring, 2013). One intriguing possibility is that even if individual updates are brief and mundane, continuously receiving fragments of personal information can result in ambient awareness of what is going on in the lives of people who post them. Science writer Clive Thompson was the first to propose how ambient awareness can develop in the context of public social media sites, such as Facebook and Twitter (Thompson, 2013). Ambient awareness can be defined as awareness of social others, arising from the frequent reception of fragmented personal information, such as status updates and various digital footprints, while browsing social media. “Ambient” emphasizes the idea that the awareness develops peripherally, not through deliberately attending to information, but rather as an artifact of social media activity. Central to this definition is that browsing social media is sufficient for awareness to develop, even in the absence of directed communication.

Prior research has looked into existing social networks, which afford directed communication (e.g., Facebook; Lampe, Ellison, & Steinfield, 2006), making it difficult to single out the contribution of mere browsing. Several scholars have considered ambient awareness, also referred to as *peripheral* or *pervasive awareness*, and its potential role in relational maintenance (Lampe et al., 2006, Resnick, 2001, Zhao et al., 2011) and organizational knowledge exchange (Dimicco et al., 2008, Leonardi and Meyer, 2014, Zhao et al., 2011). However, the construct has been discussed primarily in theoretical terms and described qualitatively, without being empirically assessed.

In the present research, we sought to establish an operational definition of ambient awareness, grounding it in relevant notions from computer-mediated communication (CMC) and psychology and to provide empirical data on primary questions related to its occurrence and functions.

## 2 Theoretical background

In face-to-face encounters, people naturally develop awareness of others by picking up on non-verbal cues. Co-workers who share the same office, for example, get a sense of each-other's daily moods and activities. Co-presence enhances communication by increasing familiarity and providing people with information. In mediated communication there is no physical presence, but a sense of social presence (i.e., quality of “being there”) and awareness can nevertheless emerge (Tu and McIsaac, 2002, Walther and Bazarova, 2008).

We expect that browsing social media posts can also contribute to a sense of awareness of the people who post them (ambient awareness). A major difference from prior work is that browsing social media is a passive, non-directed activity. In contrast, the majority of research on social presence has focused on active, interpersonal communication (e.g., Gunawardena and Zittle, 1997, Walther, 2007). Since browsing is often done distractedly, ambient awareness is rather a product of automatic social processes, such as spontaneous inferences (Ambady and Rosenthal, 1992, Uleman et al., 2008), than of deliberately trying to get to know a person, for example through active, communication, information seeking, or mentalizing. We know from psychological research that people form impressions about social others after very brief exposure to minimal content, even without intention or awareness of doing so (Uleman et al., 2008; for a review). Person-judgments are spontaneous and ubiquitous and it is therefore likely that they occur during browsing. In addition to specific impressions, the mere exposure to people's posts might lead to greater familiarity (Bornstein, 1989, Moreland and Zajonc, 1982).

### 2.1 Prevalence and content of ambient awareness

Whether ambient awareness indeed develops on public social media sites, remains to be established. In qualitative studies of enterprise social media, that is, company-intern social media, people have reported experiencing ambient awareness towards their colleagues (Zhao et al., 2011). Although informative and compelling, these subjective accounts do not provide evidence as to whether awareness is actually present. That is, it is not clear whether people are indeed aware of their online contacts or merely experience a sense of awareness, but will not be able to recognize individual members of this network. Similar problem is reflected in research, where feature use (e.g., reading comments) is considered a proxy for ambient awareness, but ambient awareness itself is not measured (e.g., Leonardi & Meyer, 2014). Another problem is that enterprise social media are different from public social media, in that people use them in a work-related context, usually with the intention to get to know or keep in touch with their colleagues. Such clearly defined context and purpose of use are not necessarily present on public social media sites, such as Twitter and Facebook, where use motivations and network composition are far more diverse. Lastly, most social media platforms offer ways of active communication (e.g., private chats and messaging), which makes it difficult to claim that any increase of awareness and familiarity is due to mere browsing (ambient awareness).

To gain insight into whether ambient awareness can develop in the relative absence of extensive, one-to-one communication, we focused on the microblogging site Twitter, where content is restricted to 140 characters and usually broadcasted to large audiences, rather than directed towards specific individuals. Thus our first research questions are:

**RQ1a**. Do people experience ambient awareness from browsing a microblogging site (Twitter), in the relative absence of one-to-one communication?

**RQ1b**. Is ambient awareness just a general sense of knowing, or does it involve recognizing individuals who are known primarily through social media?

Provided that people are indeed able to gain awareness of social others based on social media exposure, it is important to assess what kind of information they gain. The content of social information is crucial for understanding its consequences.

**RQ1c**. What specific information do social media users have about their online-only contacts?

### 2.2 Media use and relationship duration

We further set out to explore how ambient awareness relates to media use. Network size and frequency of use influence the likelihood of stumbling upon the posts of a particular user and is therefore relevant to ambient awareness. According to the theory of electronic propinquity (Walther & Bazarova, 2008), a sense of closeness in mediated communication develops more readily when people have experience with the medium they are using. Ambient awareness should therefore be higher for experienced social media users. We consider duration and frequency of social media use as indicators of experience.

**RQ2a**. What are the effects of network size and media use on the general experience of ambient awareness?

Whether one would develop awareness for a specific individual is likely influenced by how frequently one stumbles upon information about this person. Awareness should therefore be related to frequency of reading a person's posts (passive communication). While active communication can be expected to contribute to ambient awareness, important in our conceptualization is that active communication is not imperative and that awareness can be develop in its absence. Relationship duration is another relevant factor. The social information processing theory (Walther, 1996) would predict that extended periods of time and interaction are needed for awareness to develop, whereas psychological theories on impression formation (Uleman et al., 2008) would suggest that short exposure is sufficient.

**RQ2b**. How is ambient awareness of individuals influenced by passive exposure to content, active communication, and relationship duration?

### 2.3 Role in interpersonal relationships and information exchange

Enhancing awareness in mediated environments has been associated with positive effects on relationships in both personal (Cornejo et al., 2012, Ito, 2005, Liechti and Ichikawa, 2000, Romero et al., 2007) and professional context (Dourish and Bellotti, 1992, Gross et al., 2005, Liechti and Ichikawa, 2000). Similarly, social media activity can help people form awareness of their online network in a subtle, unobtrusive way.

Some evidence for the relational significance of ambient awareness comes from qualitative studies on enterprise social media. Employees who use enterprise social media have described developing ambient awareness of their colleagues, which in turn had a positive impact on relationships and information sharing (Dimicco et al., 2008, Ehrlich and Shami, 2010, Zhao et al., 2011). A recent study showed that participants who followed a person's activity on an enterprise networking site were more satisfied with subsequent information transfer from this person (Leonardi & Meyer, 2014).

According to the idea of ambient awareness, people pick up on information about social others while browsing, which resembles informal communication. Informal communication contributes to establishing common ground (e.g., Duck, Rutt, Hoy, & Strejc, 1991); Zhao et al., 2011), thereby making it easier to approach a person (Ellison, Steinfield, & Lampe, 2011). Apart from serving as a conversation starter, information about people's hobbies and profession can serve as an indication of what they are knowledgeable about. Ambient awareness can thus help social media users identify potential sources of information (Leonardi, 2015).

Understanding the role of ambient awareness in relational maintenance and information exchange is beyond the scope of this paper. However, determining whether browsing social media influences perceptions of approachability and provides knowledge of competencies is an important first step.

**RQ3a**. Does ambient awareness contribute to perceptions of approachability?

**RQ3b**. Do social media users develop awareness of their online-only contacts' hobbies and interests, including professional interests?

To address these research questions, we conducted two surveys among users of the microblogging site Twitter and developed a Twitter Network Survey procedure to assess awareness of specific individuals in participants' online network. Twitter was chosen because of the large proportion of strangers and weak acquaintances in personal networks, which allowed us to minimize the effects of prior acquaintanceship and alternative means of communication. Furthermore, content on Twitter is primarily in the form of brief posts, ambient awareness can be studied in the relative absence of more extensive forms of communication.

## 3 Study 1

### 3.1 Methods

#### 3.1.1 Twitter network survey procedure

Participants provided informed consent and temporary access to their Twitter account information. We displayed a list of 100 randomly selected people they follow on Twitter and asked them to classify as many as possible and at least 50 into (a) people they encounter primarily on Twitter (Twitter-only contacts); (b) people they encounter outside of Twitter; (c) non-human, that is, corporate accounts, brands, promoter, spam, or other automated services; (d) unknown, in case they could not at all recognize the account.

A questionnaire followed, in which we assessed participants' experience of ambient awareness, along with social media use. For the second part of the questionnaire, we displayed individual profiles (name, Twitter handle, and a profile photo) of people, whom the participants had previously classified as Twitter-only contacts. Displaying one profile at a time, we asked participants whether they knew the targets at least somewhat or not at all. When a target was at least somewhat known to the participant, a number of questions about this particular target followed. The presentation of targets (maximum 17) stopped after the participant was able to recognize and respond to questions about 5 targets. At the end, participants provided basic demographic information. They were debriefed and reimbursed. Simultaneously, we used the authentication provided by the participants to request their public data from Twitter's API. Additional Twitter data were collected through separate API requests and manual coding of profiles.

#### 3.1.2 Participants

The survey was conducted online. US citizens were recruited from an online panel (tellwut.com) and reimbursed according to the panel's standards (2$). Of the 233 initial respondents, 17 were excluded because of failing an attention check, two dropped out of the questionnaire, and one was excluded because of having an account in Twitter for less than half a week prior to the study. The final sample consisted of 213 participants (56% women), with 49% being between 18 and 34 years, 36% between 35 and 54, and 15% over 54 years. The majority of participants were employed (41%), self-employed (11%), or looking for work (6%). Homemaker was another majorly represented category (16%), followed by students (9%), unable to work (9%), and retired (6%). After excluding one outlier, the average network size was 427 (*SD* = 608; *Mdn* = 135) and the average duration of Twitter was 3.5 years (*SD* = 2).

#### 3.1.3 Materials

**General ambient awareness**: **Experience**. The experience of ambient awareness towards the network in general was assessed with a single item: “It is possible that when using Twitter, you develop awareness of the people whose updates you follow. Even if individual updates are short and mundane, together they might give you an idea of the person who posts them - what they are like, what they do, etc. Do you experience such general awareness of the people in your Twitter network and to what extent?” It was rated on a continuous scale from 1 (not at all) to 10 (to a great extent). The definition of ambient awareness used in the item was based on Thompson(2013) and refined in a qualitative pretest. In the pretest, we started with a more detailed description, featuring examples. Following participants' feedback that the described phenomenon is sufficiently clear without the additional clarifications and examples, we shortened the definition.

**General ambient awareness**: **Number of people**. Participants were asked to estimate roughly how many people they have gotten to know through posts and status updates. The answer scale ranged from 1 (several people) to 5 (almost everyone) and included a sixth, non-applicable, option.

**Awareness of individual targets**. During the survey procedure, we displayed a maximum of 17 people they followed on Twitter and asked participants whether they recognized individual profiles. This was assessed with a single item: Are you familiar with this person, ranging from 1 (not at all familiar) to 5 (very familiar). More specific questions followed for the targets who were identified as being at least somewhat familiar (answers 2 through 5).

**Network**, **media use**, **and demographics**. Relationships on Twitter are asymmetric, that is, a user can follow somebody's activities without being followed back, networks consist of (a) followers, that is, people following a user or and (b) friends, people the user follows. Being interested in the awareness users have of the people they followed, we used the latter as an index of network size. Time since registering on Twitter (in months) was used as a measure of duration. The frequency of Twitter use and general social media use were assessed on a 7-point scale ranging from 1 (*once a year or less*) to 7 (*several times a day*). Basic demographic information (gender, age, employment status) was collected. Age was measured on a categorical scale (18–24 years, 25–34 years, 35–44 years, 45–54 years, 55 years and over).

**Information categorie**s. The information participants had for each target was assessed with a checklist of common person-information categories (e.g., hobbies and interests, major life events) and asked the participants to select all categories that represent what they have gotten to know about each target. For expertise awareness, we showed lists of common recreation activities and professional sectors and asked participants to select the ones that describe the given target's hobbies and profession, respectively. All checklist variables (information categories and expertise awareness) included the options other (open-ended), not sure, and no idea.

**Approachability**. Participants reported the extent to which they find a target to be “approachable (friendly)” on a continuous scale from 1 (not at all) to 10 (extremely). The term friendly was added to clarify that approachability refers to the target's personality (i.e., warmth, friendliness) rather than availability (i.e., having enough time).

**Attention check**. Towards the end of the questionnaire we included a modified version of an instructional manipulation check (Oppenheimer, Meyvis, & Davidenko, 2009), to see whether the participants were attentive.

### 3.2 Results

#### 3.2.1 Ambient awareness (RQ1)

The majority of participants reported moderately high levels of awareness for people in their Twitter network (*M* = 5.65, *Med* = 6, *SD* = 2.09), indicating that experiencing ambient awareness was not uncommon in a diverse sample of Twitter users. To the question of estimating how many people they have gotten to know mainly through social media, the majority of responses (69%) were between few and more than several people, but less than half of the network. Awareness was not only a general experience. The majority of people (80%) were able to recognize at least 5 targets within a maximum of 17.

About half of the presented profiles (46%) were recognized, which is a substantial number, considering the large network sizes in the sample. For a selection of people identified as at least somewhat familiar, we asked participants whether they know each person outside of Twitter. A large number of people were only known through Twitter (75%). Together, these findings strongly suggest that ambient awareness can develop based on microblogging content.

#### 3.2.2 Ambient awareness of individual targets

Using the Twitter Network Survey procedure, we selected individual people from participants' own networks (targets). Targets with close relationship to the participants (i.e., family, close friends, and friends; 11%), were excluded from the analyses involving awareness. Of the remaining targets, 14% were identified as *very familiar*, 20% as *familiar*, 37% as *somewhat familiar*, and 28% as *not entirely unfamiliar*.

#### 3.2.3 Information about individual targets

**Fig. 1:**
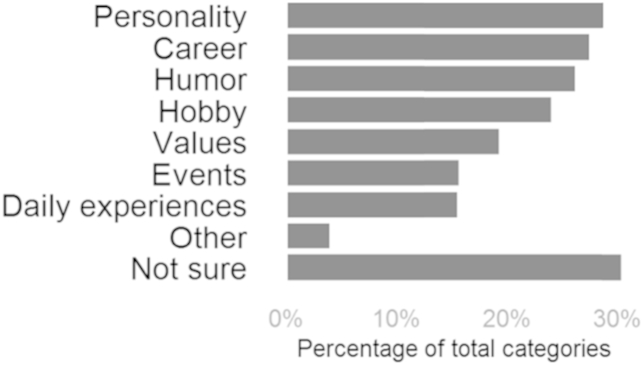
Distribution of information categories in Study 1. Checklist measure; multiple categories per target were possible.

Participants were able to report the kind of information they had encountered about individual members of their network. The checklist measure allowed for multiple information categories per target. Participants reported an average of two information categories per target. The category other was used only 2% of the time, which led us to conclude the list of categories was sufficiently comprehensive. The most commonly reported categories were information about the target's personality, career, humor, and hobbies (Fig. 1).

One type of information with particular relevance to information processes is expertise awareness, that is, awareness of who knows what in one's network. Our study included a measure, where participants indicated what a target's hobbies and profession were. The options *no idea* and *not sure* were available. Frequency analyses revealed that expertise awareness was common in the present sample. Some knowledge of targets' hobbies, profession, or both was reported for 67% of the targets.

#### 3.2.4 Demographic, network, and media use variables (RQ2)

Linear models were used to assess whether general ambient awareness and awareness of individual contacts varied across demographic groups and network characteristics. In separate models, each awareness variable was regressed on gender, age (as continuous; centered), frequency of use, network size, and time since registering on Twitter. Originally, we intended to include Twitter use as predictor, but the measure was highly skewed: 74% of all participants indicated using Twitter several times a day, which was the highest scale point. We therefore included the more normally distributed general social media use instead. Full models including all interactions were tested and whenever a simple model was not significantly different from or superior (higher Adjusted R-squared) to its complex counterpart, the simpler model is reported.

Model summaries can be seen in Table 1a. Frequency of media use was associated with both indexes of ambient awareness. Participants who used social media more frequently reported experiencing ambient awareness to a greater degree and were more likely to recognize the profiles of people from their Twitter network. Network size was negatively associated with the likelihood of recognizing individual contacts, but not with the experience of general ambient awareness. That is, people's general experience of ambient awareness did not seem to depend on the size of their network but people with large networks recognized fewer individual members of their network.

### 3.3 Discussion

The results of this study show that people experience a sense of ambient awareness towards their online network. More importantly, they were able to recognize and report information about individual people in their network, whom they know only through the microblogging platform Twitter. This awareness of individual online contacts suggests that ambient awareness is not only an illusory feeling, but that people are indeed aware of what is going on in their network. Consistent with the theory of electronic propinquity, ambient awareness was higher for frequent Twitter users. There was also an effect of age, such that older participants reported higher ambient awareness. Lastly, network size did not relate to the general experience of awareness, but seemed to be negatively associated with awareness of individual contacts.

Overall, Study 1 offers valuable insights into ambient awareness. However, it is a first, exploratory study and we cannot be certain whether the observed patterns are reliable. Furthermore, there were certain limitations. The survey procedure did not allow for precisely calculating the proportion of recognized targets, because the survey stopped after 5 targets were recognized as being at least somewhat familiar. Another problem was the scale for measuring Twitter use, which resulted in a highly skewed variable.

We therefore conducted a second study to strengthen the conclusions of Study 1 and address some of its limitations. The study kept mostly identical in order to provide additional support for the exploratory findings of Study 1.

## 4 Study 2

### 4.1 Methods

#### 4.1.1 Procedure

The procedure was identical to Study 1, except for how we presented individual profiles at the second stage of the questionnaire. Each participant saw 20 profiles for which they had to indicate whether they recognize them. From the recognized profiles, three were randomly selected and participants received additional questions, similar to those in Study 1.

#### 4.1.2 Participants

Recruitment and reimbursement were the same as in Study 1. Of the 212 respondents, 64 were excluded: 63 for failing the attention checks and one for having an account in Twitter for less than half a week prior to the study. The final sample consisted of 148 participants (68% female), 36% employed, 30% homemaker, 20% self-employed or looking for work, unable to work, student, or retired (11%, 6%, and 7%, respectively). The mean age was 41 years (*SD* = 12). After excluding one outlier, the mean network size was 519 (*SD* = 634; *Mdn* = 217) with 4 years (*SD* = 2) average duration of Twitter use.

#### 4.1.3 Materials

**Ambient awareness**. General ambient awareness and number of people for whom it is experienced were measured with the same questions used in Study 1. For awareness of individual targets, we used 8 items (see Table 2). The items were rated on a continuous scale, using a slider with anchor points 1 = not at all and 7 = extremely. The internal consistency was moderately high (Chronbach's alpha = 0.84) and the scale was treated as unidimensional.

**Network**, **media use**, **and demographics**. We changed the assessment of Twitter use in Study 2 to hours per week spent on the site to avoid the ceiling effect from Study 1. Age, which was a categorical variable in Study 1, was measured in years. All other variables remained unchanged.

**Communication and relationship duration**. Passive communication was measured by asking participants how frequently they read tweets from the given person, from 1 (never) to 7 (all the time). Active communication was measured with two items, one asking how frequently the participants interacted with the target and one asking how frequently the target interacted with the participant, answered on scales from 1 (never) to 7 (all the time). We additionally assessed relationship duration by asking participants for how long they have been following a given target on a scale from 1 (less than a week) to 5 (more than a year).

### 4.2 Results

#### 4.2.1 Ambient awareness

The average general awareness reported in Study 2 was 6.32 (*SD* = 2.25). The majority of people reported experiencing ambient awareness for between few and more than several people (75%), but less than half of the network. These patterns were similar to what we observed in Study 1.

Due to the revised survey procedure in Study 2, we were able to calculate the likelihood of recognizing individual contacts. The mean likelihood was 64% (*SD* = 38), which again is fairly high considering the large networks of the participants.

#### 4.2.2 Ambient awareness of individual targets

As in Study 1, we excluded targets with close relationship to the participants (i.e., family, close friends, and friends; 2%). In Study 2, we included an additional 8-item measure to assess ambient awareness of individual targets (Table 2). The average ambient awareness of targets known only through Twitter was moderately high (*M* = 4.51, *SD* = 1.05).

#### 4.2.3 Information about individual targets

**Fig. 2:**
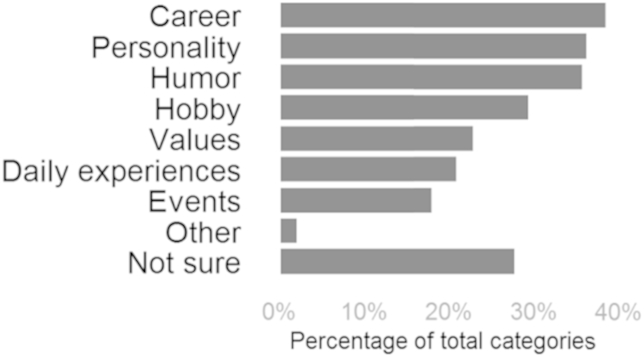
Distribution of information categories in Study 2.

Frequencies of reported information categories can be seen in Fig. 2. The most common categories were similar to those in Study 1. It should also be noted that in both studies, the category *not sure* was chosen frequently, which is in line with the idea that ambient awareness can be vague as well as specific.

#### 4.2.4 Demographic, network, and media use variables (RQ2)

The analysis strategy was the same as in Study 1. Model summaries can be seen in Table 1b. Consistent with what we found in Study 1, frequency of Twitter use was positively associated with both indexes of awareness. Network size was negatively correlated with awareness of individual contacts but not with the general experience of awareness. There was a small effect of duration of use and an interaction between age and network size. However, these effects were not consistent across studies, indicating that they are likely not robust or even spurious. We do not interpret these effects and conclude that network size and frequency of use were the only factors with consistent effects across the two studies.

The effects of relationship duration and types of communication were investigated on the level of individual targets. Due to the nested nature of the data (multiple targets per participant), multi-level models were conducted with random intercepts for participant and all factors of interest as fixed effects. Ambient awareness of the target was regressed on passive communication, active communication with target, active communication with participant, and relationship duration. All variables were standardized. As can be seen in Table 3, only the frequency with which participants read the target's posts (passive communication) and interacted with the target (active communication with target) emerged as significant predictors. Our data support the claim that ambient awareness arises on the basis of frequent exposure to bits and pieces of information (passive communication). Relationship duration had no significant effect on ambient awareness, suggesting that awareness is not strictly dependent on extended period of interaction.

#### 4.2.5 Perceptions of approachability (RQ3a)

**Fig. 3:**
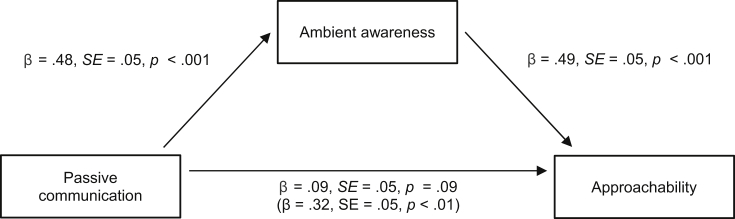
The mediating role of ambient awareness on perceptions of approachability.

The frequency of reading a target's posts was positively associated with perceptions of approachability (β = 0.32, SE = 0.05, *p* < 0.01). Our data indicate that ambient awareness mediated the relationship. The frequency of reading posts was positively related to ambient awareness (β = 0.48, SE = 0.05, *p* < 0.01) and ambient awareness was positively related to perceptions of approachability (β = 0.53, SE = 0.05, *p* < 0.01). To test for mediation, we regressed perceptions of approachability on both frequency of reading posts and ambient awareness. As can be seen in Fig. 3, the relationship between ambient awareness and approachability remained significant while controlling for reading posts, whereas the relationship between reading posts and perceptions of approachability was reduced, as compared to the direct relationship. Although our design was correlational the pattern suggests mediation and offers support for the idea that browsing social media results in ambient awareness, which has a positive impact of perceptions of approachability.

#### 4.2.6 Expertise awareness (RQ3b)

As in Study 1, expertise awareness was assessed by asking participants whether they can indicate the areas of the target person's hobbies and profession. They were also able to select *not sure* and *no idea* if that were the case. Again, having some knowledge of hobbies and profession was common. Knowledge of targets' hobbies, profession, or both was reported for 85% of the targets. Information of hobbies and profession reveal what a person is knowledgeable about and can help social media users identify who knows what in their network and thus locate potential sources of information.

## 5 General discussion

The aim of this research was to explore the idea that the passive browsing of social media timelines, increases the awareness that users have of their online networks (ambient awareness). It has been speculated but not previously demonstrated that reading updates on social media can result in ambient awareness, that is, familiarity with people within an online network. We conducted two surveys and found that people experienced moderately high levels of ambient awareness towards their network on the microblogging site Twitter and were able to report specific knowledge of people they follow on Twitter but had not met in real life.

Our research provides evidence for a central aspect of ambient awareness, which has not been explicitly addressed in prior research. Namely, that awareness in social networks can develop peripherally, from fragmented information and in the relative absence of extensive one-to-one communication. Focusing on Twitter allowed us to demonstrate that microblogging updates are sufficient for ambient awareness to develop.

Prior qualitative work has shown that people report ambient awareness (e.g., Zhao & Rosson, 2009), but it was not clear whether they become only aware of some very active network members or develop a feeling for a large proportion of their network. Most participants estimated having ambient awareness for more than several people but less than half members of their network. This suggests that although ambient awareness is not present for each online contact, it is also not limited to a small number of very active network members.

We took the assessment of ambient awareness a step further by developing a Twitter network survey procedure through which we selected individual people our participants followed on Twitter. Even though the majority of participants followed hundreds of people, they were able recognize individual members of their online-only contacts and report specific information about them.

Contrary to the idea that ambient awareness develops over extended periods of time, neither duration of Twitter use nor relationship duration predicted awareness. The general experience of ambient awareness was not influenced by network size, showing that people with large online networks can still experience a sense of knowing their social media contacts. However, they were less likely to recognize and report awareness of individual contacts, presented during the survey.

The frequency of media use was positively associated with ambient awareness. Frequency of use can be seen as indicator of experience, in which case the relationship is consistent with one of the predictions of electronic propinquity theory, namely that communicators who have experience with a given medium would benefit more from its use. Frequent use also supposes higher exposure to content, which allows for a sense of awareness to build up from fragmented information. The importance of frequent exposure is also seen on an individual level, where the ambient awareness of specific target was predicted by how often the participant received updates from the target (passive communication).

Most commonly reported was knowledge of personality, humor, hobbies, and career. The prevalence of personality and humor awareness is in line with the well-documented spontaneity of trait inferences (Uleman et al., 2008). Information about hobbies and career reveals what people are interested in and knowledgeable about. Apart from serving as topic for conversation, this information can allow social media users to gain awareness of who knows what in their network (Leonardi, 2015).

### 5.1 Limitations and future research

The present research is descriptive and largely exploratory. Although we provide converging data from two studies, additional research is needed to validate and further understand the effects we observed. We identified potential antecedents and moderators of ambient awareness, but the data were correlational. Future research can adopt methods that allow for a closer inspection of underlying processes.

The research relied on self-reported information regarding participants' own networks. While the method contributed to the ecological validity of the research, it posed restrictions to extent to which the accuracy of responses can be determined. One question is whether the participants' responses indeed reflected their actual impressions of the targets, as opposed to being formed on the spot, when participants were asked to report them. Future studies could use constructed materials and multiple judgments to definitively resolve these issues.

### 5.2 Implications

Online networks are unprecedented in size and structure. Their vastness and diversity create a potential for gaining enormous relational and information benefits (Donath, 2007), but pose a serious challenge to traditional relational maintenance strategies (Tong & Walther, 2011). As with other forms of ambient contact (e.g., Ito, 2005, Romero et al., 2007), ambient awareness is envisioned as a cognitively efficient process contributing to relational communication. Many have discussed the potentially negative effects of frequent media use when it serves as recurring distraction or leads to information overload. Seeing ambient awareness as cognitively efficient is not necessarily at odds with these findings. Rather, the efficiency stems from ambient awareness developing without cost or effort beyond what is already invested in browsing, which does not imply that browsing social media is in itself an efficient process.

One way in which awareness relates to social processes is through providing people with information about others. Such information can serve as a basis of first impressions and result in a sense of familiarity, both of which can make a target appear more approachable. This idea is supported by our data. In addition to perceptions of familiarity and approachability, ambient awareness can make it easier to approach others by providing topics for conversation. For example, somebody's social media activity can reveal that the person is an avid Joy Division fan or has recently visited a tropical island, both of which can easily serve as conversation starters. On an even more practical side, picking up on cues that somebody is stressing over an upcoming deadline is a good indication that this person might not be able or willing to respond promptly if approached.

The relational effects of ambient awareness can be linked to informational processes, because the relationship between an information seeker and source is essential to information exchange. A recent study demonstrated that connecting to a person on social media facilitated subsequent information transfer from this person (Leonardi & Meyer, 2014). The authors turn to ambient awareness to explain the link between browsing and facilitated social interaction, but do not specifically assess ambient awareness. By demonstrating that ambient awareness mediated the relationship between the frequency of reading microblogging updates and perceptions of approachability, we complement their findings and offer further support for the proposed process.

As discussed earlier, online network provide abundant valuable information, but finding efficient ways to navigate them and successfully locate information is a challenge. We found that ambient awareness involves information about hobbies and profession. Such information can enable social media users to develop a cognitive map of who knows what in their online network. Awareness of who knows what offers a potential solution to the problem of locating valuable information without needing to post public or widely shared requests. The role of expertise or knowledge awareness (who knows what) in information processes has been discussed widely in the context of collaborative work and knowledge exchange in organizations (e.g., Engelmann, Dehler, Bodemer, & Buder, 2009), but not with regard to public social media. The potential of bridging these lines of inquiry is substantial, as networks on public social media are virtually unlimited and far more diverse than organizational or other professional networks.

## 6 Conclusion

Computers and mobile devices are with us at every step of our daily lives (Vorderer, 2015). Social media and networking sites broadcast bits of information about every member of our increasingly large and diverse online networks. Often meaningless in isolation, these bits can easily be seen as random noise and clutter. Without questioning the potentially problematic effects of being permanently connected, we focused on how this incessant contact enhances our digital lives. This research is a first step towards understanding the intriguing construct of ambient awareness. We demonstrate that browsing social media and frequently encountering various social information allows social media users to gain awareness of what is going on in the lives of people in their online network. The efficacy, scope, and functionality of ambient awareness are yet to be established. We provide evidence that browsing microblogging updates is sufficient for awareness to develop and highlight ways in which it can help bring about relational and informational benefits of online networks.

## Figures and Tables

**Table 1:** Relationship between ambient awareness, demographics, and network variables.

	a. Study 1		b. Study 2	
	General ambient awareness	Ambient awareness of individuals	General ambient awareness	Ambient awareness of individuals
Gender	0.01 (0.07)	0.03 (0.07)	0.01 (0.08)	0.04 (0.08)
Age	0.10 (0.06)	0.20 (0.07)^∗∗^	−0.08 (0.08)	0.03 (0.08)
Network Size	−0.01 (0.07)	−0.31 (0.07)^∗∗^	0.08 (0.09)	−0.41 (0.08)^∗∗^
Duration	−0.03 (0.07)	0.03 (0.07)	0.17 (0.08)^∗^	0.16 (0.08)^∗^
Media use^a^	0.43 (0.07)^∗∗^	0.18 (0.07)^∗∗^	0.38 (0.09)^∗∗^	0.29 (0.08)^∗∗^
Network Size × Media use	0.02 (0.09)		−0.04 (0.10)	
Duration of use × Media use	0.03 (0.07)		0.07 (0.09)	
Gender × Media use	−0.08 (0.06)		−0.03 (0.08)	
Age × Media use	0.14 (0.07)^∗^		0.04 (0.11)	
Network Size × Duration of use	−0.21 (0.08)^∗^		−0.10 (0.10)	
Gender × Network Size	0.21 (0.07)^∗∗^		−0.05 (0.10)	
Age × Network Size	−0.07 (0.07)		0.30^∗∗^ (0.09)	
Gender × Duration of use	−0.12 (0.06)		−0.11 (0.09)	
Age × Duration of use	0.01 (0.07)		−0.08 (0.09)	
Gender × Age	0.09 (0.07)		0.04 (0.08)	
Observations	212	144	212	144
Adjusted R^2^	0.23	0.21	0.14	0.19
F Statistic (df)	5.22^∗∗^ (15; 196)	3.53^∗∗^ (15; 128)	7.78^∗∗^ (5; 206)	7.67^∗∗^ (5; 138)

Note. General ambient awareness is awareness towards the network in general; Awareness of targets is the average awareness of individual targets (maximum 17 per participant in Study 1 and 20 per participant in Study 2).

^∗^*p* < 0.05; ^∗∗^*p* < 0.01.

a General social media use in Study 1 and Twitter use in Study 2.

**Table 2:** Target awareness: ambient awareness of individual online-only contacts.

Items	*M*	*SD*
**Target Awareness Scale** (**Chronbach's Alpha** = 0.**84**)		
I feel like I know what {Name} is like as a person.	4.37	1.58
{Name}’s tweets allow me to get to know him/her at least somewhat.	4.72	1.34
{Name} is a complete stranger to me.^a^	3.61	1.76
{Name}’s is a person, I would be able to find a topic to talk about.	5.16	1.33
I have no idea what {Name} would be like in real life.^a^	3.88	1.68
I know what {Name} might be knowledgeable about.	5.06	1.42
I am aware of {Name}'s profession or professional interests.	5.14	1.59
I have an idea of what {Name}'s hobbies are.	4.24	1.69

Note. {Name} was substituted with the name and username of individual Twitter contacts.

a Reverse-coded items.

**Table 3:** Model summary of multi-level model of the effects of communication and relationship duration on awareness of individuals.

	Target awareness
Passive communication	0.36 (0.06)^∗∗^
Relationship duration	0.07 (0.05)
Active comm with T	0.21 (0.09)^∗^
Active comm with P	−0.03 (0.08)
Observations	352
Log Likelihood	−432.30

Note: Target awareness is ambient awareness of individual target (average of 8-item scale).

Active comm with T = participant interacts with target.

Active comm with P = target interacts with participant.

^∗^ p < 0.05; ^∗∗^p < 0.01.
